# Does rheumatoid synovitis activity vary during the day? Evaluation with color doppler sonography

**DOI:** 10.1186/s12891-017-1450-3

**Published:** 2017-03-04

**Authors:** Hakim Lazaar, Agnes Lhoste-Trouilloud, Bruno Pereira, Marion Couderc, Sylvain Mathieu, Martin Soubrier

**Affiliations:** 1 Clermont-Ferrand Teaching Hospital, Radiology A Department, Place H. DUNANT, Clermont-Ferrand, 63000 France; 2 Clermont-Ferrand Teaching Hospital, Biostatistics Unit, Clermont-Ferrand, 63003 France; 3 Clermont-Ferrand Teaching Hospital, Rheumatology Department, Place H. DUNANT, Clermont-Ferrand, 63000 France

**Keywords:** Rheumatoid arthritis, Ultrasonography, Synovitis

## Abstract

**Background:**

Clinical symptoms of rheumatoid arthritis (RA) improve in the course of the day, as can synovitis activity, reported via doppler ultrasound (US). The aim of the study was to establish whether the Color Doppler (CD) scores of synovitis in RA changes throughout the day.

**Methods:**

In total, 27 patients with active RA, including 14 patients receiving corticosteroids were studied. US evaluation was performed twice in each patient, at 9 a.m. (T0) and after 4 p.m. (T1) on the same day by a single radiologist and using the same instrument. Overall, 30 joints were assessed, including grey scale and CD (S0 = no flow [no detectable CD)]; S1 = mild [CD <1/3 of the synovium]; S2 = moderate [CD <2/3]; S3 = pronounced [CD >2/3]).

**Results:**

In the total population, synovitis was detected more often in the evening than in the morning (39% vs. 33%, *p* = 0.02). The difference remained significant only in patients without corticosteroid administration (44% vs. 37%, *p* = 0.04). Moreover, a greater number of CD-positive joints were likewise found (S0 vs. S1 + S2 + S3) in the evening (57% vs. 51%, *p* = 0.04) in patients not receiving corticosteroids (67% vs. 41%, *p* = 0.002). More moderate (S2) and pronounced (S3) than mild (S1) synovitis was observed at T1 vs. T0 (39% vs. 24%, *p* = 0.03) in patients not receiving corticosteroids. More synovitis (40% vs 36% *p* = 0.02) in the dominant hand were found in the evening than in the morning.

**Conclusion:**

Synovitis and CD activity increase during the day in RA patients, especially in joints of the dominant hands and in patients without corticosteroids.

## Background

Rheumatoid arthritis (RA) is a chronic, destructive, inflammatory disease of the synovium. The initial symptoms of synovitis include pain and swelling of the affected joints, with stiffness predominantly in the morning. Poor outcome, which may develop at an earlier or later point in the disease progression, is accounted for by the development of bone erosions with joint destruction, possibly leading to severe long-term disabilities.

There is evidence that ultrasound (US) displays greater sensitivity than clinical examination in detecting synovitis [1, 2]. US assessment of synovitis includes grey scale (GS; a measurement of synovial thickness or hypertrophy) and color Doppler (CD) or power Doppler (PD; a measurement of synovial vascularity, that may better reflect active inflammation). PD activity has also been shown to be the best predictor of subsequent damage in the affected joint in DMARD-treated patients in remission [2]. Ultrasonography of MCPJs is an early, reliable indicator of therapeutic response in RA [3]. Clinical RA symptoms were found to be more severe in the morning, while improving throughout the day. Laboratory findings revealed a circadian rhythm for synthesis of pro-inflammatory markers, including cytokines and interleukin (IL-6) [4]. These findings suggest that synovitis activity varies in the course of the day, which may be detected by Doppler US [5]. The aim of this study was to confirm the results obtained by Seremano et al., who showed a reduction in MCP synovitis activity in 10 patients suffering from active RA but, unlike this group, by examining the MCP, PIP and MTP joints.

## Methods

### Patients

Overall, 27 patients were enrolled in the study. All patients met the 1987 American College of Rheumatology (ACR) criteria for RA [6] with active disease defined as disease activity score based on 28 joints–erythrocyte sedimentation rate (DAS28–ESR) greater than 2.6. The entire population underwent US examination in both the morning and evening in conjunction with clinical and laboratory assessments (ESR and CRP, only performed in the morning). This study is interventional and patients were informed of their right, and provided their consent to take part. Ethical approval was obtained from the local ethics committee (CPP Sud-Est VI, 21/11/2012), and the project was submitted to the Commission Nationale de l’Informatique et des Libertés (CNIL).

### Clinical assessment

Demographic data, clinical features and RA treatments were recorded. The clinical examination investigating the painful swollen joints and thus allowing DAS28 to be calculated was only performed in the morning, as it seemed unlikely that clinical synovitis would vary between morning and evening. The DAS was recorded by experienced rheumatologists blinded to the US assessment. CRP and ESR were measured in order to assess disease activity using the DAS28-ESR and DAS28-CRP.

### Ultrasonography

Ultrasound was performed twice in each patient, at 9 a.m. (T0) and after 4 p.m. (T1) on the same day by a single experienced radiologist and using the same instrument (ANTARES, SIEMENS, 2004 or IU 22, PHILIPS, 2010 using a 5–13 MHz and a 5–12-MHz linear array transducer respectively). No stand-off pad was employed. The radiologist was blinded to the clinical findings. Overall, 30 joints were scanned from the dorsal aspect, using the long axis : bilateral wrist (distal radioulnar joint, radio- and mediocarpal joint), bilateral hand (second to fifth metacarpophalangeal (MCP) joints and proximal interphalangeal (PIP) joints and bilateral forefoot (dorsal recess of the second to fifth MTP). The US assessment of synovitis included grey scale and color Doppler measurements. Color Doppler mode was applied to minimize examination time. Synovitis on grey-scale measurements was defined via synovial hypertrophy, although no scoring was performed [7]. Synovial vascularization was visualized on color Doppler and scored semi-quantitatively from 0 to 3 (S0 = inactive synovitis [no detectable Doppler signal inside the joint]; S1 = mild [Doppler signal involving <1/3 of the synovium]; S2 = moderate [Doppler signal involving >1/3 but < 2/3 of the synovium]; and S3 = pronounced [>2/3 of the synovium involved]) [8, 9]. Still images from all the examinations were stored on local PACS (Picture archive and communication system). The examination room temperature was recorded before each US.

Intra-observer agreement was calculated by reviewing static stored images of the second to fifth MCP and the second to fifth PIP and wrists in ten patients, in color Doppler mode with a second reading taken 3 months later.

### Statistical analysis

Statistical analysis was performed using Stata software (version 12; Stata-Corp, College Station, Tex., USA). Continuous data were presented as mean ± standard deviation (SD) values. A statistical analysis was performed in order to assess the concordance between RA synovitis measurements taken in the morning and in the afternoon on both grey scale and CD. To reflect variability between different joints in the same patient, intra-subject correlations were corrected assuming an *a priori* correlation structure for correlated measurements. The primary analysis was conducted using the marginal logistic model with estimation of the generalized estimating equation (GEE). The Wald test with robust variance estimate was employed for inference. The kappa coefficient was used to measure the radiologist’s agreement (intra-observer reliability). A *p*-value of <0.05 (two-tailed) was considered statistically significant.

## Results

Overall, 23 women and 4 men with a mean age 64.3 ± 12.4 years affected by RA present for 12.9 ± 12.3 years were included in the study. The examination was performed on 17 inpatients and 10 outpatients.

Rheumatoid factor (RF) and anti-cyclic citrullinated protein (anti-CCP) antibodies were positive in 51.8% of cases, with erosive RA present in 55.5% of cases. In total, 16 patients were receiving methotrexate at a mean dose of 12.8 ± 2.3 mg, three leflunomide, two sulfasalazine and five hydroxychloroquine. Overall, 16 patients were receiving biological therapy (abatacept: six patients; rituximab: five patients; etanercept: four patients; golimumab: the remainig one patient). In addition, 14 patients were on corticosteroid therapy at a mean dose of 7.8 ± 4.3 mg. The RA was active with a DAS28-ESR of 4.7 ± 1.3 and DAS28-CRP of 4.3 ± 1.2.

All patients taking steroid administered their treatment in the morning, around 2 h before the US scan was performed. A total of 486 joints were co-investigated clinically and by US. More joints affected by synovitis were detected by US [225/486 (46.2%)] than by clinical assessments [129/486 (27%)].

In the total study population, synovitis was detected more often in the evening than in the morning (N vs. S0123), with synovitis found in 39% of cases in the evening but only in 33% in the morning (*p* = 0.02) (Table 1). This difference remained significant in patients not being administered corticosteroid tretment (44% vs. 37%, *p* = 0.04) but was not in patients under corticosteroids (34% vs. 30%, *p* = 0.20). The increase in synovitis was significant for the MCP (55% vs. 46%, *p* = 0.02) and PIP joints (28 vs. 21%, *p* = 0.03) when considering both hands but remained significant for PIP only on the dominant hand (32% vs 19%), *p* = 0.01), when each side was oserved separately.

**Table 1:** Comparison of cumulated CD synovitis and normal synovium in all patients, in those with corticosteroids (WC), and in those without (WhC) at T0 and T1

		N compared to CD synovitis (S0123)					
		Total T0 (% of joints)		Total T1 (% of joints)		p	
		WC	WhC	WC	WhC	WC	WhC
Both sides	Wrist	48		49		0.77	
		48	49	48	51	1	0.64
	MCP	46		55		**0.02**	
		41	51	48	63	0.20	*0.06*
	PIP	20		28		**0.03**	
		16	24	22	34	0.18	*0.08*
	MTP	24		26		0.41	
		20	28	21	30	0.62	0.51
	All joints	33		39		**0.02**	
		30	37	34	44	0.20	**0.04**
Right side	Wrist	46		47		0.83	
		43	49	43	51	1	0.71
	MCP	48		58		*0.07*	
		43	54	50	67	0.34	0.10
	PIP	19		32		**0.01**	
		18	21	29	37	*0.07*	*0.06*
	MTP	20		25		0.27	
		16	25	20	31	0.54	0.35
	All joints	33		40		**0.02**	
		29	36	35	46	0.19	**0.04**
Left side	Wrist	51		52		0.81	
		52	49	52	51	1	0.75
	MCP	44		52		0.11	
		39	48	46	58	0.28	0.23
	PIP	20		23		0.55	
		14	27	16	31	0.78	0.56
	MTP	27		26		0.96	
		23	31	23	30	1	0.94
	All joints	34		38		0.33	
		31	38	33	42	0.59	0.40

*p*-values are based on marginal models estimated by GEE and considering an adjustment on corticosteroid therapy (yes/no)

*﻿p*-values in bold type indicates < 0.05

*p*-values in italic type indicates between 0.05 and 0.10

WC: patients with corticosteroid therapy (*n* = 14)

WhC: patients without corticosteroid therapy (*n* = 13)

T0: before 9 am; T1: at 4 pm

N: normal synovium

S0: inactive synovitis: no detectable Doppler signal inside the joint

S1: mild synovitis: Doppler signal involving <1/3 of the synovium

S2: moderate synovitis: Doppler signal involving >1/3 but <2/3 of the synovium

S3: pronounced synovitis: Doppler signal involving >2/3 of the synovium

There was more active synovitis (S123) than inactive synovitis (S0) in the evening than in the morning (57% vs. 51%, *p* = 0.04), though this difference proved only significant in patients not receiving corticosteroids (67% vs. 41%, *p* = 0.002) (Table 2). The difference was observed in the PIP of both hands, though only in patients not receiving corticosteroids (68% vs. 36%, *p* = 0.05).

**Table 2:** Comparison of synovitis without doppler signal (S0) and with Doppler signal (S1 + S2 + S3) in all patients, in those with corticosteroids (WC), and in those without (WhC), at T0 and T1

		CD synovitis S0 compared to S123					
		Total T0 (% of joints)		Total T1 (% of joints)		p	
		WC	WhC	WC	WhC	WC	WhC
Both sides	Wrist	54		58		0.37	
		50	58	50	65	0.98	0.21
	MCP	57		66		0.08	
		43	68	46	82	0.55	*0.06*
	PIP	47		60		**0.02**	
		72	28	52	66	0.49	**0.001**
	MTP	38		35		0.83	
		45	32	29	40	0.19	0.41
	All joints	51		57		**0.04**	
		50	51	45	67	0.59	**0.002**
Right side	Wrist	49		50		0.55	
		39	58	39	60	0.43	0.75
	MCP	52		65		0.10	
		33	68	43	83	0.41	0.12
	PIP	57		60		0.37	
		80	36	50	68	0.27	**0.05**
	MTP	54		33		0.10	
		67	46	27	38	*0.06*	0.77
	All joints	52		55		0.46	
		47	56	41	67	0.51	0.12
Left side	Wrist	59		64		0.33	
		59	58	59	70	0.98	0.21
	MCP	62		66		0.41	
		55	68	50	80	0.93	0.26
	PIP	37		60		*0.07*	
		62	21	56	62	0.84	**0.02**
	MTP	25		37		0.30	
		31	20	31	43	1	0.19
	All joints	49		59		**0.04**	
		52	47	50	68	0.97	**0.01**

*p*-values are based on marginal models estimated by GEE and considering an adjustment on corticosteroid therapy (yes/no)

*p*-values in bold type indicates < 0.05

*p*-values in italic type indicates between 0.05 and 0.10

WC: patients with corticosteroid therapy (*n* = 14)

WhC: patients without corticosteroid therapy (*n* = 13)

T0: before 9 am; T1: at 4 pm

S0: inactive synovitis: no detectable Doppler signal inside the joint

S1: mild synovitis: Doppler signal involving <1/3 of the synovium

S2: moderate synovitis: Doppler signal involving >1/3 but <2/3 of the synovium

S3: pronounced synovitis: Doppler signal involving >2/3 of the synovium

The level of active synovitis (S1 vs. S23) was greater in the evening than in the morning in patients not receiving corticosteroids when considering all joints in both hands (38% vs. 24%, *p* = 0.03) (Table 3), all joints of the dominant hand (41% vs. 28%, *p* = 0.04) or the MCP of the dominant hand (59% vs. 33%, *p* = 0.01).

**Table 3:** Comparison of mild CD synovitis (S1) and moderate to pronounced CD synovitis (S2 + S3) in all patients, in patients with corticosteroids (WC), and in those without (WhC), at T0 and T1

		CD synovitis S1 compared to S23					
		Total T0 (% of joints)		Total T0 (% of joints)		p	
		WC	WhC	WC	WhC	WC	WhC
Both sides	Wrist	12		17		0.44	
		15	9	20	15	0.56	0.49
	MCP	39		54		*0.07*	
		45	36	44	45	0.99	**0.03**
	PIP	50		48		0.85	
		62	29	62	62	0.96	0.33
	MTP	26		11		0.31	
		40	11	14	40	0.35	0.98
	All joints	31		39		0.10	
		38	24	37	38	0.93	**0.03**
Right side	Wrist	11		16		0.68	
		29	0	29	8	0.97	0.98
	MCP	33		59		**0.01**	
		25	37	42	66	0.25	**0.02**
	PIP	33		43		0.22	
		50	0	63	31	0.48	0.52
	MTP	33		11		0.26	
		50	17	0	17	0.32	0.89
	All joints	28		41		**0.04**	
		38	20	40	42	0.56	**0.03**
Left side	Wrist	13		19		0.59	
		18	15	21	18	0.53	0.95
	MCP	45		49		0.61	
		35	46	50	35	0.51	0.28
	PIP	75		53		0.35	
		67	60	50	67	0.44	0.68
	MTP	14		10		0.97	
		0	25	0	0	0. 80	1
	All joints	34		36		0.76	
		29	34	37	29	0.77	0.50

*p*-values are based on marginal models estimated by GEE and considering an adjustment on corticosteroid therapy (yes/no)

*p*-values in bold type indicates < 0.05

WC: patients with corticosteroid therapy (*n* = 14)

WhC: patients without corticosteroid therapy (*n* = 13)

T0: before 9 am; T1: at 4 pm

S1: mild synovitis: Doppler signal involving <1/3 of the synovium

S2: moderate synovitis: Doppler signal involving >1/3 but <2/3 of the synovium

S3: pronounced synovitis: Doppler signal involving >2/3 of the synovium

Subgroup analysis on patients with/without methotrexte (MTX) and with/without biotherapy were performed, showing no statistical differences between morning and evening assessment of synovitis (N vs S0S1S2S3), CD-positive synovitis (S0 vs. S123) or pronounced synovitis (S1 vs. S23).

The examination room was 23.8 ± 0.7°C in the morning and 25.4 ± 0.9 in the evening. Intra-observer reliability values were 0.94 to 1 for MCP joints and 0.86 to 1 for PIP joints.

## Discussion

Our study shows that the number and activity of joints affected by synovitis increase throughout the day. More joints affected by synovitis were found in the evening than in the morning. The same has applies to synovitis activity but in patients not receiving corticosteroids. In these patients, more active synovitis was observed in the evening than in the morning in the PIP of the dominant hand, with higher intensity for the PIP and MCP.

Conversely, a report by Semerano et al. [5] reported on a diurnal variation in the PDUS signal from MCP joints in 10 patients with active RA, which was higher in the morning than in the afternoon or evening, along with the disappearance of Doppler activity from some joints in the evening. The authors suggested that this variation appears to mirror the circadian variation in the joint stiffness and pain wharacterizing RA.

Semerano et al., however, studied only MCP joints in the morning, the joints in which we found least variation in synovitis activity, apart from the increase in grade 2 and 3 right-hand MCP synovitis. Additional studies are thus required to explain these inconsistent results. Th observed difference could also be explained by the administration of corticosteroids, which were shown to reduce the variation in synovitis intensity in our study. The administration of corticosteroids was not detailed in the study by Semerano, in which the first echography was carried out between 7 a.m. and 10 a.m. In our study, patients had taken their medication proior to undergoing echography.

Several elements that might explain our results can be considered. The first is the fact that US is operator-dependent, although this does not appear to be the explanation, as the investigation was performed by an experienced radiologist, reproducible results.

The room temperature was higher in the evening than in the morning, which may have potentially affected the results. Some authors have reported a decrease in Doppler signal after cooling the skin [10, 11]. Ellegaard et al., however, showed that heat did not affect Doppler synovitis activity [11]. This explanation is thus rather unlikely. Further explorations are required in oreder to better understand why synovitis is found more often in the evening than in the morning.

The larger number and greater activity of synovitis joints in the dominant hand may suggest that physical activity plays a role. That said, a study invilving 29 RA patients showed no significant changes in the wrist Doppler signal after 8 weeks of exercise, though the MCP and PIP joints were not studied in this work [12].

US is an increasingly used tool in the management of RA patients. In agreement with results reported in the literature, US allowed for detecting more inflamed joints than clinical examination in our study [13]. Numerous studies have shown that PD activity of synovitis to be a predictive factor for radiological progression and relapse in patients in remission with low disease activity [14, 15].

Moreover, US was often able to detect acute synovitis in patients considered to be in clinical remission [16–18]. The US examination, however, was only performed in the dominant hand in these studies which was shown to vary most over the day according to our results [17, 18].

## Conclusions

Our results suggest the relevance of considering the time at which studies are initiated when assessing the utility of US remission compared to clinical remission [19].
